# The Yeast Forkhead Transcription Factors Fkh1 and Fkh2 Regulate Lifespan and Stress Response Together with the Anaphase-Promoting Complex

**DOI:** 10.1371/journal.pgen.1002583

**Published:** 2012-03-15

**Authors:** Spike D. L. Postnikoff, Mackenzie E. Malo, Berchman Wong, Troy A. A. Harkness

**Affiliations:** Department of Anatomy and Cell Biology, College of Medicine, University of Saskatchewan, Saskatoon, Canada; The University of North Carolina at Chapel Hill, United States of America

## Abstract

Forkhead box O (FOXO) transcription factors have a conserved function in regulating metazoan lifespan. A key function in this process involves the regulation of the cell cycle and stress responses including free radical scavenging. We employed yeast chronological and replicative lifespan assays, as well as oxidative stress assays, to explore the potential evolutionary conservation of function between the FOXOs and the yeast forkhead box transcription factors *FKH1* and *FKH2*. We report that the deletion of both *FKH* genes impedes normal lifespan and stress resistance, particularly in stationary phase cells, which are non-responsive to caloric restriction. Conversely, increased expression of the *FKHs* leads to extended lifespan and improved stress response. Here we show the Anaphase-Promoting Complex (APC) genetically interacts with the Fkh pathway, likely working in a linear pathway under normal conditions, as *fkh1Δ fkh2Δ* post-mitotic survival is epistatic to that observed in *apc5^CA^* mutants. However, under stress conditions, post-mitotic survival is dramatically impaired in *apc5^CA^ fkh1Δ fkh2Δ*, while increased expression of either *FKH* rescues APC mutant growth defects. This study establishes the *FKH*s role as evolutionarily conserved regulators of lifespan in yeast and identifies the APC as a novel component of this mechanism under certain conditions, likely through combined regulation of stress response, genomic stability, and cell cycle regulation.

## Author Summary

Throughout human evolution, one question has remained constant: can we live forever? We are continuously bombarded with products, diets, and exercise regimens that supposedly add years to our life. Is there an alterable program, whether genetic or environmental, that can be tweaked to increase longevity? Medical advances have led to a dramatic increase in average lifespan over the last century. However, the maximum human lifespan has curiously remained constant. Recent research indicates that in many organisms a genetic program exists to control lifespan. The conservation of this genetic lifespan program extends into yeast where numerous longevity genes have been isolated and characterized. Interestingly, mutations that reduce genomic instability, glucose utilization, or oxidative damage extend lifespan in multiple organisms. Here we characterize one such set of genes, the FOXOs. In animals, these genes increase lifespan and suppress tumors, but have yet to be associated with longevity in yeast. By confirming that these genes play a similar role in yeast, we provide a tool to identify downstream factors triggered by the FOXOs, a feat which has not yet been accomplished in other systems. Considering the conservation of these factors, it is likely that our discoveries in yeast will be directly applicable to research into human cancer and aging.

## Introduction

The evolutionarily conserved insulin-signaling pathway plays a critical role in multiple cellular processes [1]–[4]. Perhaps most important is the decisive role it plays in cellular, and organismal, survival. This pathway must be tightly regulated, as overactive insulin-signaling leads to increased survival of cells that would otherwise be shunted down the apoptotic pathway. This occurs by increased repression of stress response, pro-apoptotic, and DNA repair genes, thereby increasing the proliferative capacity, and oncogenic potential of these cells. Although the lifespan of damaged cells is increased under these conditions, this situation increases the probability that the organism will die prematurely due to disease onset. On the other hand, reduced insulin-signaling relieves repression of the stress response, cell cycle arrest and DNA repair pathways, increasing cell maintenance capacity and survival. It is now clear that there is a link between diabetes and cancer [5]–[7], diseases associated with the insulin-signaling pathways, highlighting the importance of understanding the precise activity of this pathway.

The insulin-signaling phosphorylation cascade activates AKT, which targets cellular factors that switch metabolism from catabolic to anabolic reactions, favoring growth and reproduction over maintenance and repair [8]. Major AKT targets include the forkhead box O family (FOXO) transcription factors (reviewed in [9]–[14]). The FOXOs are believed to serve diverse rolls in longevity determination and tumor suppression in metazoans from nematodes to humans. The FOXOs integrate signals from energy, growth factor and stress signaling cascades to regulate cell differentiation, cell-cycle progression, apoptosis, autophagy, DNA-damage repair, and scavenging reactive oxygen species. FOXO proteins have been shown to interact with multiple cofactors that mediate their activity through posttranslational modifications. Phosphorylation, ubiquitination, methylation, and acetylation regulate transcription factor efficiency and nuclear shuttling (reviewed in [9], [10]). Specifically, nutrient (insulin) and growth factor signals lead to cytosolic sequestering and ubiquitination of the FOXOs, targeting them for degradation; conversely, internal reactive oxygen species (ROS), DNA damage sensing and starvation signals can cause nuclear shuttling, and transcription factor activity. Thus, a dynamic and complex molecular network controls FOXO protein function, yet specific downstream targets remains speculative.

*Saccharomyces cerevisiae* is often utilized to elucidate regulation of fundamental eukaryotic mechanisms. However, the individual deletion of any of the four Forkhead box orthologs does not affect lifespan [15], suggesting a lack of functional conservation. However, two of the orthologs, *FKH1* and *FKH2*, show genetic redundancy, as deletion of both genes is necessary to alter growth, cell morphology and gene transcription phenotypes [16]–[20]. Further evolutionary conservation for *FKH1* and *FKH2* is suggested by their requirement for ROS-induced cell cycle arrest [18], and cell cycle regulation through the regulation of both G1 and G2/M gene clusters [17], hallmarks of the human FOXO genes.

The Fkh1/2 regulated CLB2 gene cluster [17] encodes genes required for Anaphase-Promoting Complex (APC) activity (*APC1*, *CDC5*, *CLB2*, and *CDC20*), as well as APC targets (*CLB2*, *CDC5*, *CDC20* and *IQG1*) [21]–[23]. The APC is a highly conserved multi-subunit ubiquitin-protein ligase (E3) that promotes mitotic progression and G1 maintenance by targeting cell cycle regulators, such as the securin Pds1 and the cyclin Clb2, for proteasome-dependent degradation [21], [23], [24]. The APC has been demonstrated to be critical for regulating genomic stability, and longevity in yeast and higher eukaryotic organisms [25]–[30]. In yeast, mutation to multiple APC subunits decreases replicative lifespan (RLS; measures mitotic longevity) and chronological lifespan (CLS; measures post-mitotic survival), while over-expression of *APC10* increases RLS [29]. In mice, mutations to the APC regulator BubR1, a component of the spindle checkpoint, lead to premature aging defects [27], [31]. Consistent with this, we and others have provided evidence that the yeast APC plays a role in stress response by possibly targeting proteins that block proper stress response for degradation [28], [32], [33].

Our data supports a model where *FKH1* and *FKH2* function is evolutionarily conserved with higher eukaryotic FOXO proteins with regards to lifespan and oxidative stress resistance. We show that the *FKH*s are required for increased stress resistance and survival in response to severe caloric restriction (cultures maintained in water). Importantly, we identify the APC as a potential target of the *FKHs* under normal conditions, while functioning cooperatively under stress conditions.

## Results

### *FKH1* and *FKH2* encode redundant longevity determinants

**Figure 1:**
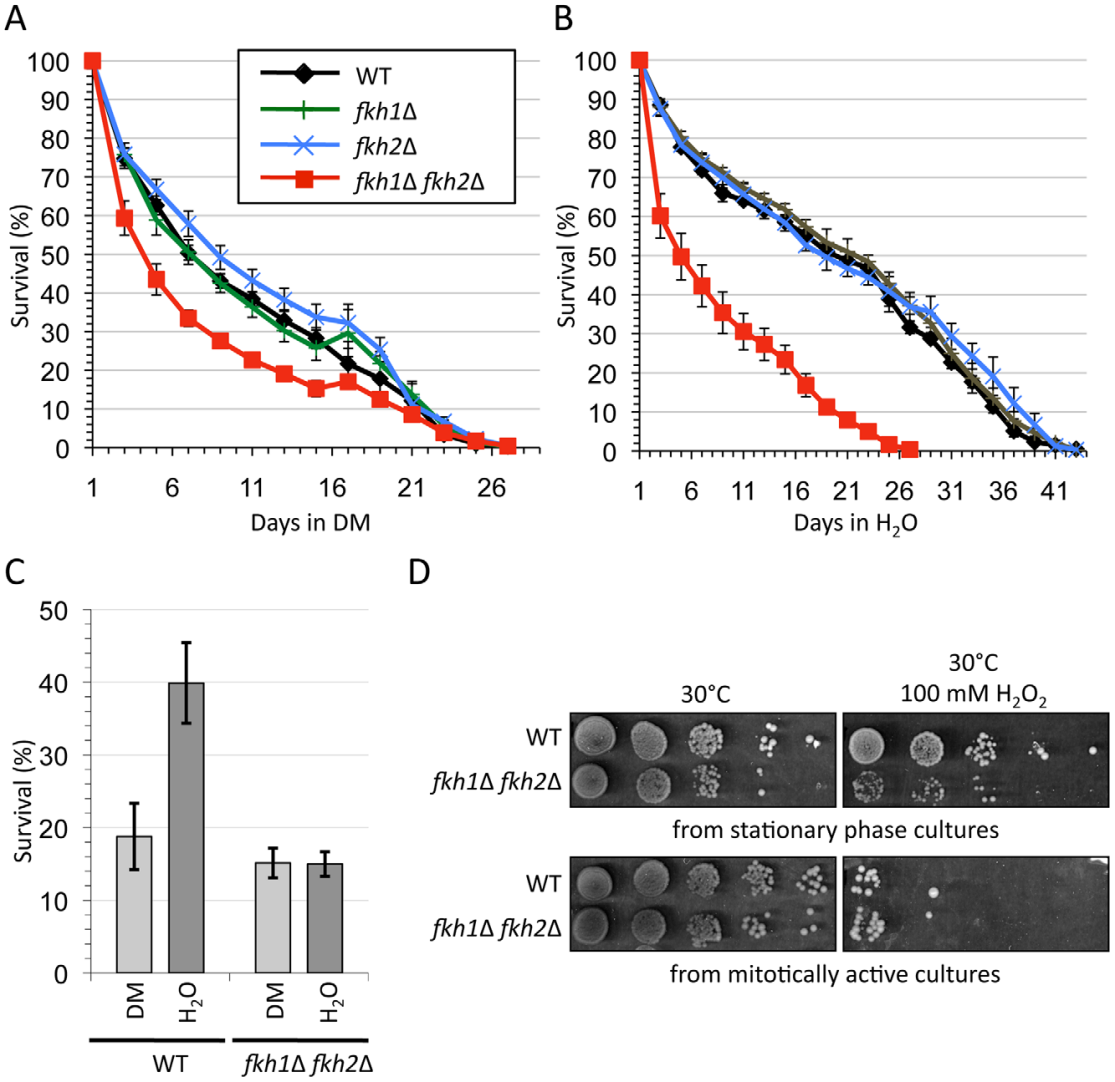
*FKH1* and *FKH2* encode redundant determinants of lifespan and stress response. (A) and (B) The cells shown were grown in CM (2% glucose) to stationary phase. The cells were either left in (A) depleted CM (DM), or (B) washed and resuspended in H_2_0 for the remainder of the experiment. Colony counts were performed every other day throughout the experiment. The day when the colony count peaked was considered Day 1. Standard error is shown for at least 3 replicates. (C) Acute oxidative stress in stationary phase cells. WT and *fkh1Δ fkh2Δ* cells were grown to day 5 of stationary phase with maintenance in either DM or H_2_O. 100 mM H_2_O_2_ was then added to one half of each sample and incubated for 60 minutes at 30°C. Diluted cells were then plated on YPD media and the colony forming units were counted. Survival was determined by dividing the colony forming units following H_2_O_2_ treated by untreated samples. Standard error of at least 3 replicates is shown. (D) Chronic oxidative stress in mitotically active and stationary phase cells. WT and *fkh1Δ fkh2Δ* cells from overnight log phase cultures or day 5 stationary phase cultures were treated with 100 mM H_2_O_2_ at 30° for 1 hour, as above, then spot diluted onto YPD plates in the absence of stress. The plates were grown at 30°C for 3 days.

FOXO transcription factors regulate processes involved in the homeostasis of metazoan cells and tissues with the net outcome being lifespan extension and tumor suppression, yet many of the downstream targets remain unknown [9]–[14]. The budding yeast *Saccharomyces cerevisiae* is a powerful tool used to elucidate genetic and molecular mechanisms mediating many cellular processes, but independent deletion of the four yeast forkhead box protein encoding genes (*FKH1*, *FKH2*, *HCM1*, *FHL1*) does not alter CLS [15]. However, Fkh1 and Fkh2 have been shown to be phenotypically redundant, as they are required for M/G1 progression and cell cycle arrest in response to hydrogen peroxide [18]. These characteristics lead us to examine the role of both Fkh1 and Fkh2 in the regulation of yeast lifespan. We investigated the *FKHs* using the RLS assay, a measure of the mitotic lifespan of individual cells, finding that deletion of either individual *FKH* gene had no effect on RLS, as reported for CLS [15]. Double mutant cells could not be assayed using RLS due to their flocculent phenotype (data not shown). Therefore, we investigated the potential of the *FKHs* in regulating CLS, a measure of metabolic activity in post-mitotic stationary phase cells [34]. In cultures maintained in depleted complete media (DM), we also observed that single deletion of the *FKH* genes did not impair CLS. Deletion of both *FKH1* and *FKH2*, on the other hand, reduced CLS (Figure 1A), with mean (50%) survival reached by day 8.5 for WT cultures, day 11 for *fkh2Δ* cultures, day 8 for *fkh1Δ* cultures, and only day 4 for *fkh1Δ fkh2Δ* cultures.

Controversy exists as to whether higher eukaryotic FOXOs, downstream targets of nutrient/insulin signaling, are contributing factors to caloric restriction-induced lifespan extension [35]–[39]. To examine whether the yeast *FKHs* play a role in caloric restriction, we examined the CLS of the *FKH* mutants by maintaining the post-mitotic cultures in distilled H_2_O. Water is believed to act as a form of severe caloric restriction (SCR) that simulates the low-nutrient environment that yeast in the wild would most likely encounter [40], [41]. Maintenance in H_2_O extended the mean survival of WT, *fkh1Δ* and *fkh2Δ* cultures to 19–21 days, while little change was observed in *fkh1Δ fkh2Δ* cultures with a mean survival of 5 days (Figure 1B). The lack of response in *fkh1Δ fkh2Δ* cultures maintained in H_2_O suggests that Fkh1 and Fkh2 play a redundant role in SCR-induced lifespan extension.

Although Fkh1 and Fkh2 have not previously been associated with longevity in yeast, they have been linked with stress response in mitotically active cells [18], which is associated with an evolutionarily conserved role in long lifespan [42]–[44]. To address whether the Fkhs' role in longevity is a manifestation of their involvement in stress resistance in post-mitotic cells, we treated WT and *fkh1Δ fkh2Δ* day 5 stationary phase cells maintained in either H_2_O or DM with 100 mM hydrogen peroxide (H_2_O_2_) for 1 hour (Figure 1C). WT day 5 stationary phase cultures exhibited increased resistance to H_2_O_2_ when maintained in water compared to DM. However, this effect was nullified in *fkh1Δ fkh2Δ* cultures, indicating that the Fkh proteins are required for stress resistance during stationary phase. A plate assay confirmed that stationary phase cells exhibit increased stress response compared to mitotically active cells, and that deletion of *FKH1* and *FKH2* diminishes this effect (Figure 1D).

### The Fkh proteins are present during stationary phase and have a higher nuclear content in H_2_O

**Figure 2:**
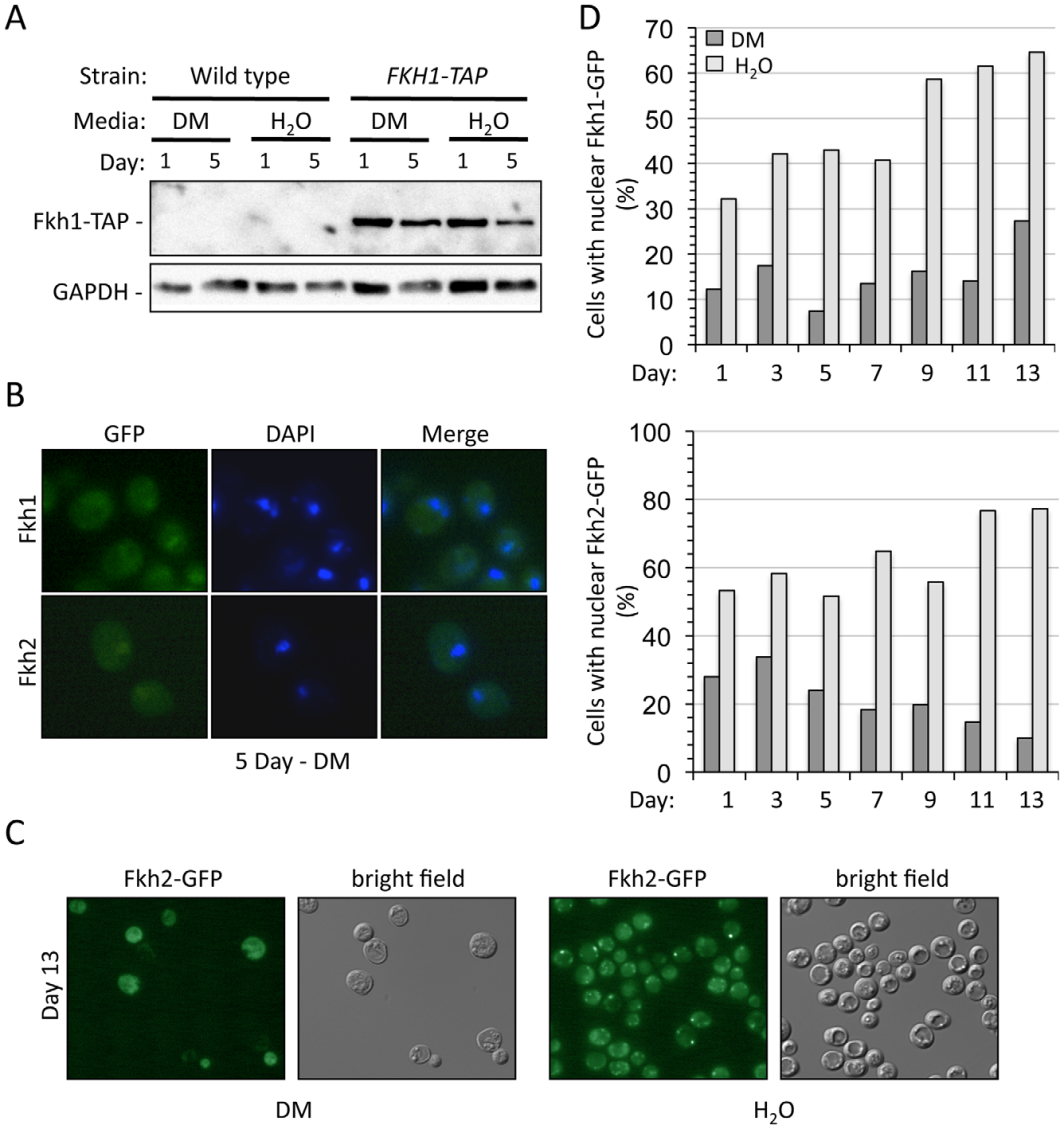
The Fkh proteins are present in the nucleus of stationary phase cells. (A) Cells expressing endogenously TAP-tagged *FKH1* were grown to stationary phase and either left in DM or transferred to H_2_O for the remainder of the experiment. Samples were removed on days 1 and 5 for Western analysis using antibodies against the TAP epitope, or GAPDH as a load control. Fkh2-TAP was also observed in stationary phase cells (data not shown). (B) Cells expressing endogenously tagged *FKH1*- or *FKH2*-GFP were grown to day 5 stationary phase while maintained in DM. Cells were observed to harbor both Fkh1 and Fkh2 nuclear staining. (C) Day 13 stationary phase cells expressing Fkh2-GFP were imaged, showing reduced nuclear staining in cells maintained in DM. (D) The percentage of nuclear localized Fkh1-GFP or Fkh2-GFP was determined as cells aged in either DM or H_2_O.

To further assess the role of the *FKHs* in normal and stressed post-mitotic lifespan, the endogenous genes encoding Fkh1 and Fkh2 were C-terminally TAP (tandem affinity purification)-tagged, with protein levels analyzed as cells aged in DM media and H_2_O (Figure 2A). Fkh1-TAP was indeed expressed as cells aged during stationary phase in DM and H_2_O. Fkh2-TAP was also observed in aging stationary phase cells, but at much lower levels (data not shown). Fkh1-TAP levels appear slightly lower in day 5 stationary phase cells compared to day 1, with very little difference between H_2_O and DM. Nonetheless, Fkh1 and Fkh2 proteins are expressed in post-mitotic cells.

Next, endogenous C-terminal GFP-tagged Fkh1 and Fkh2 were analyzed in aging cells to determine cellular localization. In day 5 stationary phase cultures maintained in DM, GFP fluorescence was observed to be nuclear in many cells (Figure 2B). When Fkh1-GFP and Fkh2-GFP were monitored in progressively aging cells in DM and in H_2_O, we observed that a larger proportion of the Fkh protein remained nuclear in H_2_O compared to DM. An example is shown in Figure 2C, where day 13 stationary phase cells appear healthier, with a larger proportion of nuclear Fkh2-GFP when maintained in H_2_O (Figure 2C). The percentage of cells harboring nuclear Fkh1-GFP or nuclear Fkh2-GPF was consistently observed to be greater when the cells were maintained in H_2_O compared to DM (Figure 2D). This suggests the presence and nuclear localization of the Fkhs may be necessary for normal CLS and stress resistance, especially in an SCR environment.

### Increased expression of the *FKHs* enhances stress resistance, CLS, and RLS

**Figure 3:**
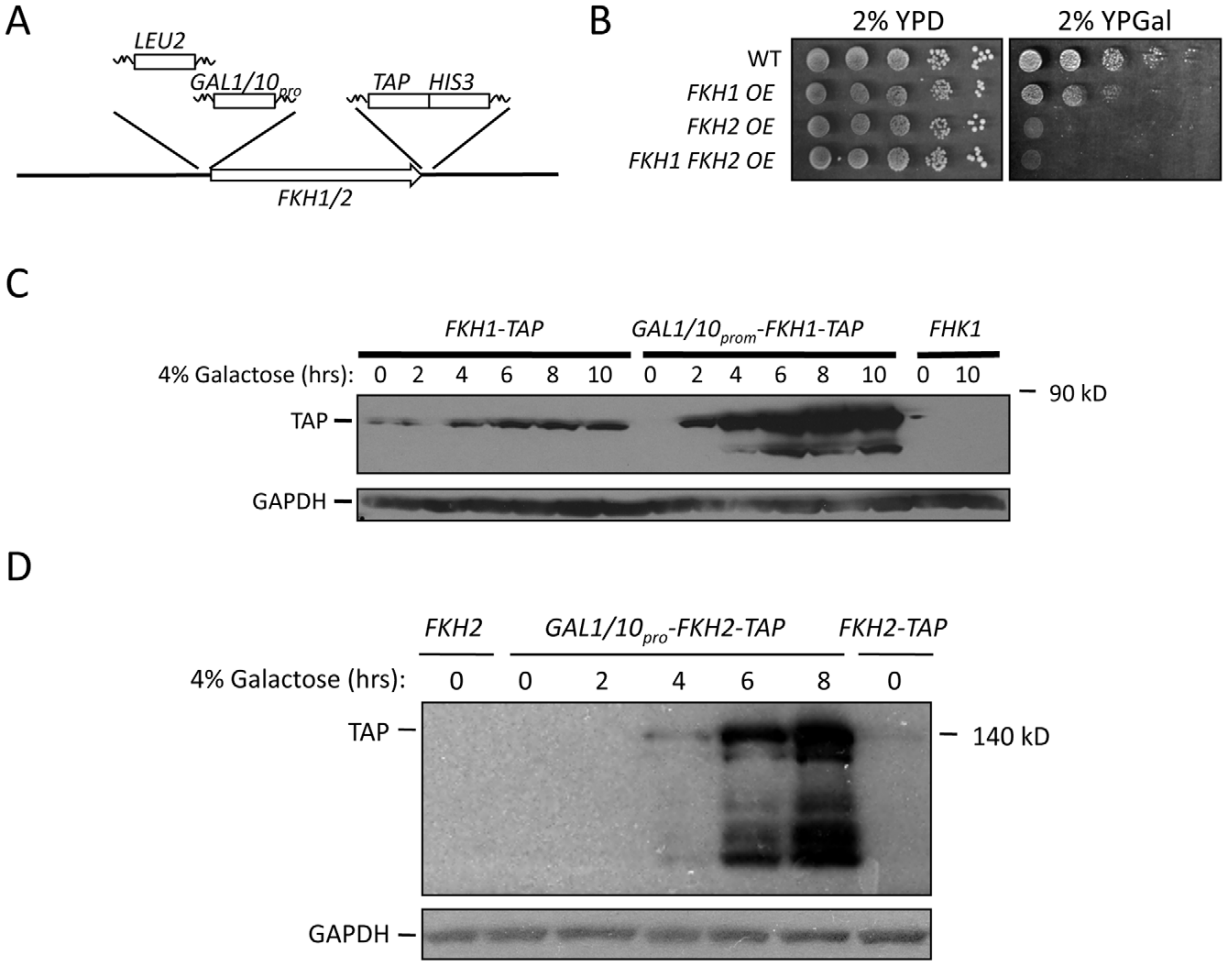
Increased *FKH1* or *FKH2* expression improves stress resistance and extends CLS and RLS. (A) Schematic representation of scheme used to integrate the *GAL1*/*10* promoter upstream of *FKH1* and *FKH2*. The *LEU2* PCR product, containing 300 basepairs of *LEU2* promoter (incorporated for selection purposes), was flanked by 60 basepairs of homology to the *FKH* promoter and to the *GAL1*/*10* promoter. The *GAL1*/*10* promoter PCR fragment was flanked by 60 basepairs of homology to the 3′ end of *LEU2* and to the 5′ end of the *FKH* gene. Cells were cotransformed with both products and selected on leu^−^ plates. Cells harboring *FKH1* and *FKH2* under the control of the *GAL1*/*10* promoter were generated by crossing the single integrated strains. (B) Cells overexpressing (OE) *FKH1* and/or *FKH2* from the *GAL1*/*10* promoter were grown overnight in 2% YPD, then spot diluted onto the plates shown. The plates were incubated at 30°C for 2 to 5 days. (C) *FKH1-TAP* OE cells were grown overnight in 2% glucose. The next day, the cells were washed and resuspended in media containing 2% galactose. Samples were taken every two hours for 10 hours for Western analysis using antibodies against TAP and GAPDH. Cells expressing *FKH1-TAP* and *FKH1* under their own promoter were used as controls. (D) *FKH2-TAP* OE cells were analyzed as described above for *FKH1-TAP* OE cells.

**Figure 4:**
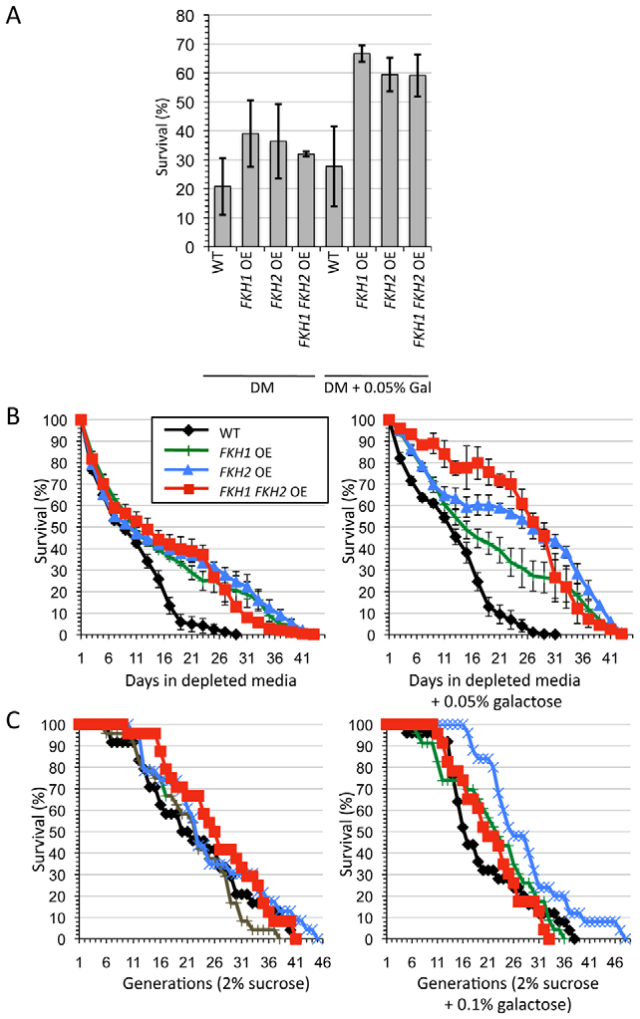
Increased expression of the *FKH* genes increases lifespan and stress response. (A) The *FKH* OE cells were grown to stationary phase, then either maintained in DM, or 0.05% galactose was added. The cells were incubated for an additional 5 days, then split, with one half treated with 100 mM H_2_O_2_ for 1 hour. The other half served as the untreated control. Following the 1 hour incubation, the cells were diluted and plated onto YPD until colony forming units formed. Survival was determined by dividing the treated cells by the untreated cells. Standard error is shown for at least 3 replicates. (B) CLS was determined for the OE strains when maintained solely in DM (left panel) or in DM supplemented with 0.05% galactose (right panel). Standard error is shown for at least 3 replicates. (C) RLS was determined for the OE strains on 2% sucrose plates or sucrose plates supplemented with 0.1% galactose. Typical results are shown.

In higher eukaryotic systems increased expression of FOXO orthologues is associated with increased longevity and stress resistance [38], [45]. Thus, we predicted that an increase in survival would be expected with the overexpression of *FKH1* and/or *FKH2*. Increased *FKH* expression was accomplished by integrating the *GAL1*/*10* inducible promoter immediately upstream of the *FKH1-TAP* and *FKH2-TAP* start sites (Figure 3A). Cells overexpressing both *FKH1* and *FKH2* were created by crossing the appropriate strains. Growth of these cells on 2% glucose was comparable to WT, but growth was diminished when *FKH* overexpressing (OE) cells were grown on 2% galactose-supplemented media (Figure 3B). Fkh1-TAP and Fkh2-TAP were weakly expressed in 2% glucose, but massively expressed after 6 hrs in 2% galactose (Figure 3C, 3D). Lower concentrations of galactose (0.05–0.1%) did not influence vegetative growth (data not shown), but did improve stress resistance and longevity (Figure 4). First, we measured the ability of 5 day stationary phase cultures maintained in DM to survive a 1 hour treatment of 100 mM H_2_O_2_. For this experiment, once cells reached stationary phase the cells were split with one sample receiving a supplement of 0.05% galactose. After 5 days, samples were removed, treated with H_2_O_2_, and then diluted onto YPD plates to determine colony forming units. Controls were cells that did not receive H_2_O_2_. The *FKH* OE cultures exhibited increased survival in the absence of galactose, with improved resistance when supplemented with galactose (Figure 4A). These observations are consistent with a role for the Fkh proteins in stress resistance during stationary phase.

If the Fkh proteins do enhance stress resistance during stationary phase, then it is likely that increased *FKH* expression may prolong metabolic activity in these cells. CLS of WT and *FKH* OE cells was measured in DM in the presence and absence of 0.05% galactose (Figure 4B). Cells were grown in 2% glucose to stationary phase, then split, with one sample receiving a supplement of 0.05% galactose. Unaltered WT cells were used as a control. We observed that the addition of 0.05% galactose increased the CLS mean lifespan (50% survival) of the unaltered WT control from approximately 8.5 to 12 days. In the absence of galactose the OE strains exhibited mean lifespans of 9.5 to 12 days. However, in the presence of 0.05% galactose, the *FKH1* OE strain experienced a 15 day mean lifespan, while *FKH2* OE strains enjoyed mean lifespans of approximately 27 days. Yeast cell lifespan can be measured in post-mitotic stationary phase (CLS), or in rapidly dividing mitotic cells (RLS). Stress resistance plays a major role in determining both CLS and RLS, however not all genes that influence CLS also influence RLS. Sir2 in yeast is a good example of this [44]. To determine whether the Fkh proteins also influence RLS, we measured RLS in the cells employed in Figure 4B using 2% sucrose as a base carbon source in the presence and absence of 0.1% galactose (Figure 4C). In the absence of galactose, RLS of all strains was relatively unchanged. Upon 0.1% galactose supplementation, *FKH2* OE cells had a markedly longer RLS. The mean lifespan of *FKH1* OE cells was also increased, but not to the same extent as the *FKH2* OE cells. We also observed increased RLS in *FKH2* OE, but not *FKH1* OE cells using 0.05% galactose (data not shown). The enhanced stress resistance and CLS observed in OE strains in the absence of galactose are not surprising as we previously documented the basal activity of the galactose promoter [33]. Our results are consistent with the Fkh proteins playing a role in responding to stress, which may indirectly lead to increased CLS and RLS. The effect appears to be greater during post-mitotic stationary phase cells, with Fkh2 perhaps playing a more pivotal role compared to Fkh1.

### The Fkh proteins and the Anaphase-Promoting Complex (APC) work together to mediate post-mitotic survival

The advantage of using yeast for genetic studies is the relative ease of identifying interacting partners for proteins and genes of interest. Thus, we sought possible downstream Fkh targets that may be involved in stress response and longevity. One possible target of the Fkh transcription factors is the Anaphase-Promoting Complex (APC). The APC is an evolutionarily conserved ubiquitin-protein ligase (E3) that targets proteins that inhibit mitotic progression and exit, as well as G1 maintenance, for ubiquitin- and proteasome-dependent degradation [21], [23]. We previously observed APC mutants to exhibit reduced CLS and RLS, while increased *APC10* expression extended RLS [29], [46]. Furthermore, APC mutants are sensitive to DNA damaging agents, and exhibit both chromatin assembly and histone modification defects [28], [33], [46]–[49]. Consistent with the APC's involvement in histone biogenesis and lifespan, we recently demonstrated that yeast cells harboring histone modification defects are subject to reduced RLS [50]. The APC appears to play an evolutionarily conserved role in lifespan, as mutations to mouse BubR1, a component of the spindle checkpoint that inhibits APC function, resulted in inappropriate APC activity and premature aging phenotypes [27]. A possible link between the APC and the Fkhs was revealed by a previous microarray analysis of *fkh1Δ fkh2Δ* cells, where *APC1* (APC subunit), *CDC5* (APC activator/target), *CLB2* (APC activator/target), *CDC20* (APC activator/target), and *IQG1* (APC target), were identified as responsive to the Fkh transcription factors [17], [21], [22]. A subsequent analysis of *CLB2* mRNA expression during the cell cycle (*CLB2* mRNA synthesis is cell cycle regulated) showed that it was defective in *fkh1Δ fkh2Δ* mutants [16]. Clb2, a mitotic cyclin that is an important activator of the APC, later becomes targeted by the APC for degradation to allow exit from mitosis [21]. To test our hypothesis that APC activity may be targeted and activated by the Fkh proteins, we created *fkh1Δ fkh2Δ* cells harboring a mutation in the APC subunit Apc5 to enable genetic analyses. *APC5* encodes an essential APC subunit (the *apc5^CA^* allele used in our studies contains a two basepair deletion at the 5′ end of the coding region, most likely creating an N-terminally truncated protein [28]; unpublished data). Cells with the *apc5^CA^* mutation grow slowly at temperatures above 36°C, which can be recovered or exacerbated by genetic alteration of negative or positive regulators, making this an excellent allele to identify *APC5* interacting partners [29], [33], [47], [49], [51].

**Figure 5:**
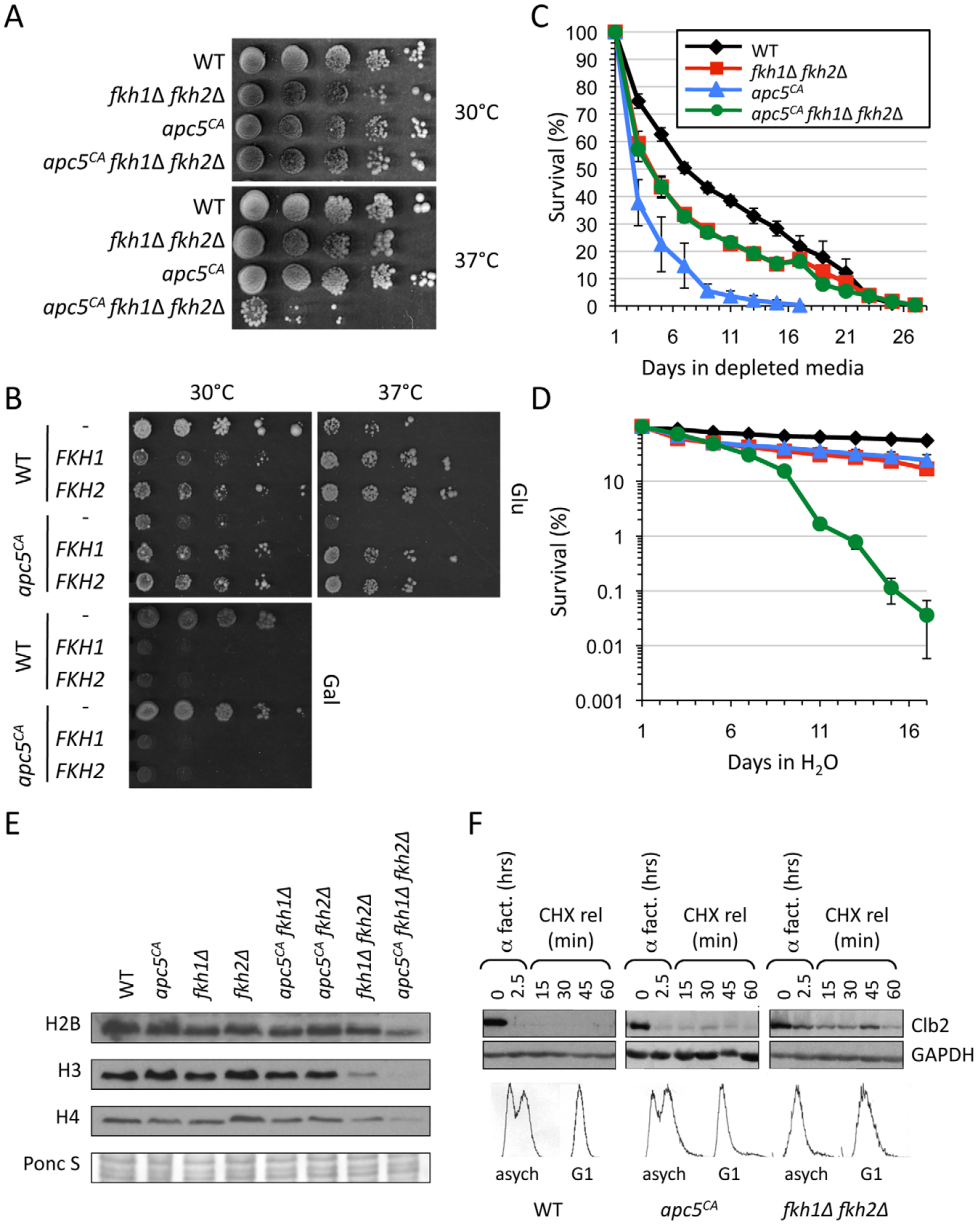
The Fkh proteins and the APC work together to promote extended CLS and stress response. (A) The *FKH* genes were deleted in *apc5^CA^* cells by multiple rounds of genetic crosses. The strains shown were grown overnight at 30°C in YPD, then spot diluted onto YPD plates and incubated at 30 or 37°C. (B) WT and *apc5^CA^* cells were transformed with plasmids expressing *FKH1* or *FKH2* from a galactose inducible promoter, or an empty vector control plasmid. Individual transformed colonies were grown overnight and then spot diluted onto SD ura- plates supplemented with either 2% glucose or 2% galactose. The plates were incubated at 30 or 37°C. (C) The mutants used above were grown to stationary phase and maintained in depleted media (DM) for the remainder of the experiment. Colony forming units were determined every other day and a survival curve was plotted. Standard error is shown for at least 3 repeats. (D) CLS was determined for the strains used above. Rather than maintenance in DM, the cells were washed once they reached stationary phase and maintained in H_2_O for the remainder of the experiment. Standard error is shown for at least 3 repeats. The experiments in (C) and (D) were started from the same cultures. (E) The panel of strains shown were grown overnight at 30°C to early log phase growth. Proteins were extracted and analyzed by Westerns to assess total histone H2B, H3 and H4 protein levels. A Ponceau S stained gel is included to shown equivalence of protein load. (F) WT, *apc5^CA^* and *fkh1Δ fkh2Δ* cells encoding *BAR1* were grown to early log phase, then arrested with 2 µg/ml α factor for 1.5 hours in pH 3.5 YPD media. Another 2 µg/ml α factor was added followed by another hour of incubation. Cycloheximide was then added with continued incubation. Samples were removed at the times shown and Clb2 protein levels were determined by Western blotting. Antibodies against GAPDH were included as a load control. The same blot was divided and used for the Clb2 and GAPDH Westerns. Samples were taken before and after G1 arrest for FACS analysis.

First, we examined the growth characteristics of *apc5^CA^ fkh1Δ fkh2Δ* mutants. Deletion of both *FKH* genes severely impaired *apc5^CA^* growth at the restrictive temperature, but not at the permissive temperature (Figures 5A). Deletion of both *FKH* genes also impaired the growth of *apc10Δ* cells and was lethal in the *apc11-13* background (unpublished data). This preliminary investigation provides evidence that the APC and the Fkhs may share a common function.

If the Fkhs and the APC do share a common function, then increased expression of the *FKHs* may restore the *apc5^CA^* temperature sensitive (*ts*) growth phenotype. Thus, we expressed plasmid borne *FKH1* and *FKH2* under the control of the *GAL1/10* promoter in WT and *apc5^CA^* cell. The cells were grown in 2% glucose supplemented media, then spot diluted onto plates containing either 2% galactose or 2% glucose (Figure 5B). At 30°C, expression of the *FKHs* on glucose-supplemented media was slightly detrimental to WT cells, but beneficial to *apc5^CA^* cells. Galactose-driven genes do have basal activity in the presence of glucose [33]. On galactose plates, overexpression of either *FKH* was toxic, as observed above (Figure 3B). At 37°C, expression of either *FKH* gene on glucose plates improved growth of both WT and *apc5^CA^* cells. We have also observed that increased *FKH1* expression suppressed the *ts* defect in additional APC mutants, including *apc10Δ*, *apc11-13*, *cdc16-1*, *cdc23-1*, *apc5^CA^ apc10Δ* and *apc5^CA^ cdc26Δ* cells (data not shown). These observations suggest that under conditions of stress, whether temperature or impaired APC activity, moderately increased *FKH* expression improves the capacity of the cell to cope.

Next, we asked whether deletion of both *FKHs* influenced *apc5^CA^* CLS. Cells expressing the different combinations of mutations were grown to stationary phase, then split, with one half resuspended in H_2_O, and the other half left in DM. Equal volumes were then plated on the days shown to generate survival curves (Figure 5C, 5D). In DM, *apc5^CA^* cells rapidly lost viability compared to WT and *fkh1Δ fkh2Δ* cells (Figure 5C). Interestingly, the triple mutant survived as long as *fkh1Δ fkh2Δ* cells. This suggests that deletion of both *FKH1* and *FKH2* is epistatic to the *apc5^CA^* allele under the conditions tested. In other words, under normal media conditions using DM, the Fkhs appear to act upstream of the APC. When the experiment was conducted by maintaining the cells in H_2_O for the duration of the experiment, a different survival profile was observed (Figure 5D). WT (20 days vs 7 days) and *apc5^CA^* (5 days vs 2.5 days) cells both responded to H_2_O conditions by exhibiting a longer mean CLS. However, *fkh1Δ fkh2Δ* cells had the same CLS in H_2_O as in DM, which was similar to *apc5^CA^* cells in H_2_O, whereas the triple mutant had a greatly reduced CLS in H_2_O. As stated above, the failure of *fkh1Δ fkh2Δ* cells to survive longer under SCR conditions, such as H_2_O, suggests that the Fkhs are required for long life under SCR conditions. The similar H_2_O CLS observed in *fkh1Δ fkh2Δ* and *apc5^CA^* cells, and the dramatically reduced H_2_O CLS in the triple mutant indicates that the Fkhs and the APC may have redundant functions under stress conditions. This contrasts with the Fkh/APC epistatic interaction that appears to occur under DM conditions. This could reflect dual roles for the Fkh proteins; as cell cycle regulators under normal conditions, and as stress response proteins under stress conditions [17], [18], [52], [53]. Lastly, these observations clearly identify another non-mitotic function for the APC. The APC has been shown to function in other non-mitotic activities, such as meiosis, quiescence, differentiation, metabolism, maintenance of post-mitotic neurons, and interestingly, memory in mice [30], [54]–[58].

In addition to controlling CLS, the APC and the Fkhs are also involved in histone metabolism, but likely through very different mechanisms. The Fkhs are redundant activators of cell cycle dependent histone expression [17]. On the other hand, histones and histone modifications are reduced when genes encoding different APC subunits, such as *APC5*, *APC9*, *APC10*, *APC11*, *CDC16*, *CDC23* and *CDC26*, are mutated [33]. The mechanism involved remains elusive, but it is likely post-transcriptional, as histone mRNAs are unaltered in APC mutants [33]. Our analysis of total histone levels in the different mutant combinations indicates that histone control is indeed through different redundant mechanisms, as histone levels are greatly reduced in the triple mutant compared to the single and double mutants (Figure 5E). However, it remains possible that the Fkhs drive histone synthesis through direct transcriptional control and indirectly via the APC.

As a direct assessment of whether the Fkhs control an APC function, we measured Clb2 stability in *apc5^CA^* and *fkh1Δ fkh2Δ* mutants. Clb2 is a B-type cyclin that is targeted by the APC for degradation in order to exit mitosis [21]. *CLB2* transcripts are also controlled by the Fkhs [16], [17]. WT, *apc5^CA^* and *fkh1Δ fkh2Δ* cells were grown to early log phase at 30°C, then arrested in G1 using α factor. The cells were determined to be arrested in G1 using microscopy (data not shown) and FACS analysis (Figure 5F). The cells were then released into cycloheximide, with samples removed every 15 minutes for protein analysis using antibodies against endogenous Clb2. In both WT and *apc5^CA^* cells Clb2 levels were decreased in G1 arrested cells when compared to asynchronous cells (Figure 5F). In *fkh1Δ fkh2Δ* cells however, the degradation of Clb2 was reduced in G1 arrested cells. However, it should be noted that *fkh1Δ fkh2Δ* cells accumulated in G1 in asynchronously grown cells, as indicated by FACS, perhaps reflecting the inability to degrade mitotic cyclins. These observations are consistent with a model where the Fkh transcription factors act in a positive manner upstream of the APC, perhaps through transcriptional activation of APC subunits, activators and targets.

### The APC and the Fkhs function in the stress response pathway

**Figure 6:**
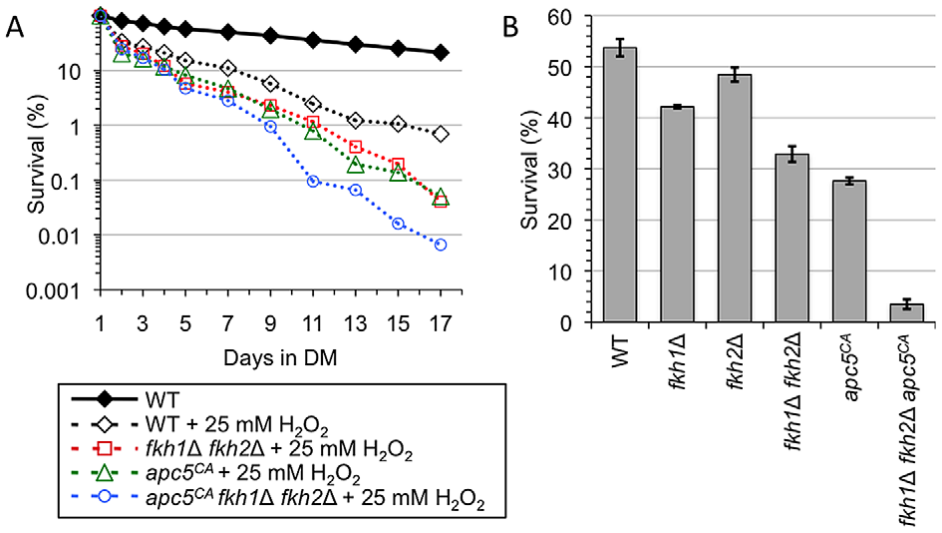
The APC and the Fkh proteins provide overlapping function to respond to oxidative stress. (A) CLS was performed using the strains shown in the presence of 25 mM H_2_O_2_. The cells were grown to stationary phase, and then H_2_O_2_ was added to the cultures. Colony forming units were determined every other day for the remainder of the experiment. Survival curves are shown. (B) The strains described above were used to determine resistance to oxidative stress. Day 5 stationary phase cells were exposed to 100 mM H_2_O_2_ for 1 hour at 30°C. Controls were not treated with H_2_O_2_. Diluted cells were then plated onto YPD plates until colonies formed. The percent survival was determined, with standard error shown for at least 3 replicates.

Since the APC is associated with maintaining lifespan in multiple systems [27], [29], [30], [31], we tested whether the APC may also be involved in oxidative stress resistance. To test this hypothesis, we conducted CLS in the presence of oxidative stress in WT, *apc5^CA^*, *fkh1Δ fkh2Δ* and *apc5^CA^ fkh1Δ fkh2Δ* cells. The cells were grown to stationary phase followed by the addition of 25 mM H_2_O_2_ (Figure 6A), with cell counts determined every other day. The results show WT cells had a reduced lifespan in response to 25 mM H_2_O_2_, while the mutants were further impaired. The lifespan of the triple mutant was dramatically reduced compared to *apc5^CA^* and *fkh1Δ fkh2Δ* cells in H_2_O_2_, as it was in water (Figure 5D). These observations provide additional evidence that the triple mutant is extremely sensitive to stress and likely perceives water as a severe stress, rather than a form of caloric restriction. To our knowledge, few mutations have been described that act in a negative manner to SCR.

**Figure 7:**
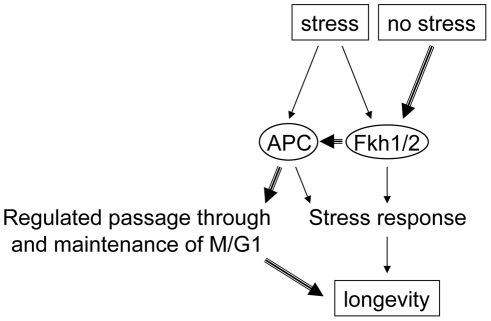
Model depicting how the APC and the Fkh proteins may interact under normal and stress conditions. Under normal conditions, the Fkh proteins play a role in activating the APC. This in turn mediates progression through mitosis and maintenance of G1. This interaction is depicted by bold arrows. Under stress conditions, both the APC and the Fkh proteins respond via different mechanisms. The Fkh proteins likely promote the expression of stress response proteins. The APC acts according to a mechanism that remains uncharacterized, but likely requires ubiquitin-dependent processes.

To investigate the roles of the APC and the Fkh proteins in stress resistance further, stationary phase cultures were treated with 100 mM H_2_O_2_ for 30 minutes at 30°C, and then plated to determine cell viability. While 53.2% of WT cells survived 100 mM H_2_O_2_, only 21.3% of *apc5^CA^* cells and 31.3% of the *fkh1Δ fkh2Δ* survived the treatment (Figure 6B). However, the *apc5^CA^ fkh1Δ fkh2Δ* mutant was dramatically impaired, with only 3.5% surviving this treatment. Taken together, our data indicates that the APC and the Fkh proteins have overlapping functions in response to oxidative stress in post-mitotic cells, opposed to the epistatic interaction observed in unstressed cells (Figure 5C, Figure 7). Consistent with an APC involvement in post-mitotic CLS, the APC is also required for stress response in post-mitotic cells.

## Discussion

This report provides evidence to support an evolutionarily conserved role for the yeast forkhead transcription factors Fkh1 and Fkh2 in lifespan determination and stress response. *FKH1* and *FKH2* act redundantly in controlling CLS and post-mitotic stress response, as deletion of both genes is required to observe CLS and stress response defects (Figure 1), as previously described for other phenotypes [16], [17]. Importantly, we show that a modest increase in *FKH1* or *FKH2* expression results in increased CLS, RLS and stress resistance (Figure 4). The Fkhs may have a dual function in cell cycle progression and in stress response. A microarray analysis initially identified such a role, as arrest of *fkh1Δ fkh2Δ* cells in G1 identified a series of genes involved in cell cycle progression, whereas the transcript profile identified in asynchronous *fkh1Δ fkh2Δ* cells was composed of stress response genes [17]. Importantly, we identify an evolutionarily conserved cell cycle regulator, the Anaphase-Promoting Complex (APC), as a potential downstream target of the Fkh transcription factors (Figure 5, Figure 6). Genetic interaction studies between APC and Fkh mutants revealed a possible division of Fkh labor. For example, the CLS of *apc5^CA^* and *fkh1Δ fkh2Δ* cells showed an epistatic interaction under normal conditions (Figure 5C), suggesting the Fkhs may be upstream of the APC. However, under stress conditions, such as maintenance in H_2_O (Figure 5D) and in the presence of 25 mM H_2_O_2_ (Figure 6A), the CLS of the triple mutant was dramatically impaired beyond any of the single and double mutants, defining a synergistic interaction. This most likely reflects a role for the Fkhs in activating the transcription of genes involved in stress response (Figure 7). Thus, under non-stress conditions, the Fkhs may play a role in driving APC activity that leads to controlled progression through mitosis and into G1. Since the APC is required for genomic stability, activating the APC would be predicted to increase the fidelity of chromosome segregation, reducing nondysjunction events, thereby increasing the potential for a healthier and longer cellular lifespan. The APC also plays a role in stress response (Figure 6B). Previously, it was shown cells lacking the Cdh1 APC activator were sensitive to multiple stresses, such as ethanol, caffeine and salt [32], and we have shown that *apc5^CA^* and *apc10Δ* cells are sensitive to UV and methylmethanesulfonate (MMS) [28], [33]. Stabilization of the APC targets Clb2 and Hsl1 also increased stress sensitivity [32], indicating the APC may activate the stress response by alleviating inhibitory signals. The Fkhs' contribution to longevity is most likely an indirect reaction to stress, occurring via at least two pathways, one involving transcription of stress genes [17], [18], and the other through driving APC activity (Figure 7). It has long been established that cells better equipped to repair damage and respond to stress stand a better chance to live a healthier and potentially longer lifespan [34], [42], [43], [59]. These observations demonstrate the evolutionary conservation of the FOXO transcription factors in yeast, which respond to stress and extend cellular lifespan.

### Fkh1, Fkh2, and severe caloric restriction (SCR)

The initial focus of this work was to determine whether the conserved yeast Fkh proteins were involved in longevity, as shown with metazoan FOXOs. Our work clearly demonstrates a need for the Fkh proteins for extended CLS and RLS. However, our work also demonstrates that the Fkh proteins are necessary for extended lifespan in response to SCR. Recently, the Rim15 stress responsive transcription factor was identified as a major mediator of SCR lifespan extension [15]. Deletion of *RIM15* blocked extended lifespan in *ras2Δ*, *sch9Δ* and *tor1Δ* strains, indicating that the phenomenon of SCR funnels through Rim15. Interestingly, although deletion of *RIM15* in the extremely long-lived *ras2Δ sch9Δ* mutant reduced lifespan under normal conditions, this strain could still respond to SCR, suggesting other factors can respond to SCR in the absence of Rim15. Our data provides the possibility that Fkh1/Fkh2 may fulfill this role. Future work will require an analysis of strains lacking *FKH1*, *FKH2* and *RIM15*.

### The APC is likely a downstream target of the stress responsive Fkh proteins

We tested the hypothesis that the Fkh proteins play a role in lifespan by contributing to APC activation. Our results support this hypothesis, as (i) low-level expression of *FKH1* or *FKH2* suppressed APC mutant growth phenotypes; (ii) deletion of both *FKH1* and *FKH2* exacerbated APC mutant histone and growth phenotypes in mitotically active cells; and (iii) deletion of *FKH1* and *FKH2* stabilized the APC substrate Clb2. Furthermore, stress resistance and lifespan in the presence of stress were markedly worse in the triple mutant compared to the single and double mutants. These results demonstrate that the APC and the Fkhs function together in both mitotic and post-mitotic cells. While *fkh1Δ fkh2Δ* cells do not respond to SCR, *apc5^CA^ fkh1Δ fkh2Δ* exhibit decreased CLS under these conditions. This suggests that H_2_O is perceived as much more than a low level stress in the triple mutant, which is consistent with the Hormesis hypothesis of aging, a theory that postulates low level stresses turn on stress defense mechanisms, leading to potenially longer life [60]. Nonetheless, these observations implicate the APC as a player in caloric restriction and stress response.

We observed that APC and *FKH* mutants interacted differently depending on the growth conditions. Figure 7 presents a model describing how this may occur. Under non-stress conditions, such as growth on YPD or maintenance of stationary phase cells in the depleted media (DM), there was little interaction (growth on YPD at 30°) or an epistatic interaction (CLS in DM). The interaction could be interpreted to define a pathway where the Fkhs act upstream of the APC. It could be as simple as the Fkhs driving the transcription of the APC subunit Apc1, and the APC activators Clb2, Cdc5 and Cdc20 [16]. This interaction is likely more complicated than direct classical epistasis, perhaps involving stoichiometric alteration of APC activators, subunits and/or substrates. The deletion of the *FKH*s in APC mutant strains may bring about a homeostatic balance between APC activity and substrate levels. On the other hand, under stress conditions, such as high temperature or the addition of H_2_O_2_, *apc5^CA^ fkh1Δ fkh2Δ* cells exhibited a synergistic interaction. This type of interaction occurs when multiple proteins drive a similar activity in a redundant manner, thus requiring that more than one mutation must occur to expose a phenotype. Both the Fkhs and the APC are required for response to stress, but likely act in very different manners. The Fkhs drive expression of many stress response genes [16], whereas the molecular mechanisms employed by the APC to combat stress may involve APC^Cdh1^, which has been found to regulate different stress responses through the degradation of substrates such as Clb2 and Hsl1 as a part of the APC's role in G1 maintenance [32]. An additional role the APC likely plays in stress revolves around histone metabolism. We have shown that APC mutants exhibit defects in histone maintenance, chromatin assembly and histone modifications [28], [33], [47]–[49], [51]. It is well established that histone modifications, such as phosphorylation of H2A/H2AX, methylation of H3 Lys79, and acetylation of H3 Lys56 and H4 Lys16, are required for recruitment of DNA repair enzymes to sites of DNA damage [61]–[65]. Therefore, under stress conditions, the Fkhs and the APC likely play separate, yet overlapping roles in ensuring the cell responds to stressful damaging agents in a positive manner.

Our studies provide a potential link between the Fkh transcription factors and the APC that ties the APC together with stress response. This provides insight into a molecular mechanism whereby the APC facilitates longevity. The evolutionarily conserved nature of our results, and the established role the APC plays in tumor development and progression, suggests that the APC may be a potential downstream target of the insulin signaling pathway. A recent report supports this scenario, as the insulin driven AKT1 kinase phosphorylates the APC^Cdh1^ substrate Skp2, an SCF component, which inhibits Skp2 degradation [66]. This enables SCF activity and promotes cell cycle progression. These studies offer the basis for further studies in understanding APC-dependent longevity.

### Conclusions

This study identifies the yeast forkhead box containing proteins Fkh1 and Fkh2 as regulators of lifespan, allowing for the characterization of upstream and downstream regulation by these factors. This may provide insight into how the highly related FOXO proteins in mammals regulate both lifespan extension and tumor suppression. Our data supports a model where *FKH1* and *FKH2* are functionally orthologous with the metazoan FOXOs, which opens the door to genetic manipulation in yeast for further exploration into the function and mechanisms controling these important metabolic and stress regulators.

## Materials and Methods

### Yeast strains and plasmids

The yeast strains used in this study are shown in Table 1. The *fkh1*Δ::*kanMX6* and *fkh2*Δ::*kanMX6* strains, obtained from the ResGen library (provided by W. Xiao, U. of Saskatchewan), were repeatedly backcrossed to our S288c background strains to generate *apc5^CA^*, *apc10Δ* and *apc11-13* congenic partners. *apc11-13* cells were a kind gift from T. Hunter (Salk Institute). Cells harboring *APC9*, *APC10* and *CDC26* deletions were acquired from W. Xiao and backcrossed repeatedly to our S288c background. *cdc16-1*, *cdc23-1* and the isogenic wild type were generously provided by D. Stuart (U. of Alberta). The C-terminal *FKH1-TAP* and *FKH2-TAP* strains were generously provided by A. Ghavidel (U. of Toronto). PCR based methods were used to TAP-tag *FKH1* and *FKH2* in various mutants. Cells expressing endogenously GFP-tagged *FKH1* and *FKH2* were obtained from Open BioSytems. The galactose inducible *FKH1-HA* and *FKH2-HA* encoding plasmids were obtained from the Research Genetics library of tandem affinity tagged plasmids purchased by W. Xiao (U. of Saskatchewan). The *GAL1*/*10* promoter was integrated upstream of the *FKH1* and *FKH2* genes by PCR-based homologous integration approach. Two sets of primers were designed for this approach. First, primers were designed to amplify the *LEU2* gene (plus 300 basepairs of the promoter) flanked on the 5′ side by 60 nucleotides of sequence homologous to the immediate promoter regions of *FKH1* or *FKH2*, and the 3′ side by 60 nucleotides homologous to the *GAL1*/*10* promoter. The second primer set was designed to amplify *GAL1*/*10* promoter flanked on the 5′ side by 60 nucleotides homologous to the 3′ end of the *LEU2* gene and on the 3′ end by 60 nucleotides homologous to the first 60 nucleotide of *FKH1* or *FKH2*. Primer sequences are available upon request. The two PCR fragments were transformed together into WT cells. Leu^+^ transformants were selected for further analysis.

**Table 1:** *Saccharomyces cerevisiae* strains used in this study.

Strain	Genotype	Source
YTH5	*MAT*α *ade2 his3Δ200 lys2Δ201 ura3-52*	Harkness *et al.* 2002
YTH6	*MAT*α *ade2 his3Δ200 lys2Δ201 ura3-52*	Harkness *et al.* 2002
YTH457	*MAT*α *ade2 his3Δ200 leu2-3,112 ura3-52 apc5^CA^*	Harkness *et al.* 2002
YTH1235	*MAT*a *ade2 his3Δ200 leu2-3,112 lys2Δ201 ura3-52*	Harkness *et al.* 2002
YTH1636	*MAT*a *ade2 his3Δ200 leu2-3,112 ura3-52*	Harkness *et al.* 2004
YTH1637	*MAT*α *ade2 his3Δ200 leu2-3,112 ura3-52 apc5^CA^*-PA*::His5*	Harkness *et al.* 2004
YTH1693	*MAT*(?)*his3 leu2 met15 ura3 apc10Δ::kanMX6*	Harkness *et al.* 2005
YTH2290	*MAT*a *his3Δ1 Δleu2 Δmet15 Δura3 fkh1Δ::kanMX6*	W. Xiao
YTH2291	*MAT*a *his3Δ1 Δleu2 Δmet15 Δura3 fkh2Δ::kanMX6*	W. Xiao
YTH2427	*MAT(?) ade2 his3 leu2 lys2*(?) *Δmet15*(?) *ura3 fkh1Δ::kanMX6*	This study
YTH2431	*MAT(?) ade2 his3 leu2 lys2*(?) *Δmet15*(?) *ura3 fkh1Δ::kanMX6 apc5^CA^*-PA*::His5*	This study
YTH2444	*MAT(?) ade2 his3 leu2 lys2*(?) *Δmet15*(?) *ura3 fkh2Δ::kanMX6*	This study
YTH2449	*MAT(?) ade2 his3 leu2 lys2*(?) *Δmet15*(?) *ura3 fkh2Δ::kanMX6 apc5^CA^*-PA*::His5*	This study
YTH2578	*MAT*a *ade2 his3 lys2*(?) *Δmet15*(?) *ura3 fkh1Δ::kanMX6 fkh2Δ::kanMX6*	This study
YTH2579	*MAT(?) ade2 his3 leu2 lys2*(?) *Δmet15*(?) *ura3 fkh1Δ::kanMX6 fkh2Δ::kanMX6*	This study
YTH2581	*MAT(?) ade2 his3 lys2*(?) *Δmet15*(?) *ura3 apc5^CA^*-PA*::His5 fkh1Δ::kanMX6 fkh2Δ::kanMX6*	This study
YTH2582	*MAT*a *ade2 his3 leu2 lys2*(?) *Δmet15*(?) *ura3 apc5^CA^*-PA*::His5 fkh1Δ::kanMX6 fkh2Δ::kanMX6*	This study
YTH3124	*MAT(?) ade2 his3 leu2 lys2*(?) *Δmet15*(?) *ura3 apc10Δ::kanMX6 fkh1Δ::kanMX6*	This study
YTH3143	*MAT(?) ade2 his3 leu2 lys2*(?) *Δmet15*(?) *ura3 apc11-13 fkh2Δ::kanMX6*	This study
YTH3346	*MAT(?) ade2 his3 leu2 lys2*(?) *Δmet15*(?) *ura3 apc10Δ::kanMX6 fkh2Δ::kanMX6*	This study
YTH3405	*MAT(?) ade2 his3 leu2 lys2*(?) *Δmet15*(?) *ura3 apc11-13 fkh1Δ::kanMX6*	This study
YTH3408	*MAT(?) ade2 his3 leu2 lys2*(?) *Δmet15*(?) *ura3 apc10Δ::kanMX6 fkh1Δ::kanMX6 fkh2Δ::kanMX6*	This study
YTH3409	*MAT(?) ade2 his3 leu2 lys2*(?) *Δmet15*(?) *ura3 apc10Δ::kanMX6 fkh1Δ::kanMX6 fkh2Δ::kanMX6*	This study
YTH3926	as YTH1235, but *FKH1-TAP::HIS3*	This study
YTH3929	as YTH1235, but *FKH2-TAP::HIS3*	This study
YTH3930	as YTH457, but *FKH1-TAP::HIS3*	This study
YTH3933	*apc10Δ::kanMX6 FKH1-TAP::HIS3* (YTH1693×3926)	This study
YTH3999	*MAT(?) apc10Δ::kanMX6 FKH2-TAP::HIS3* (YTH1693×3929)	This study
YTH4110	as YTH457, but *FKH2-TAP::HIS3*	YTH3409
YTH3409	*MAT(?) ade2 his3 leu2 lys2*(?) *Δmet15*(?) *ura3 apc10Δ::kanMX6 fkh1Δ::kanMX6 fkh2Δ::kanMX6*	This study
YTH4265	as YTH3929, but *LEU2::GAL1/10_prom_*-*FKH2-TAP::HIS3*	This study
YTH4269	*MAT*α *ade2 his3Δ200 leu2-3,112 lys2Δ201 ura3-52*	This study
YTH4315	*MAT*a *his3Δ1 Δleu2 Δmet15 Δura3 FKH1-GFP::HIS3*	Open Biosystems
YTH4316	*MAT*a *his3Δ1 Δleu2 Δmet15 Δura3 FKH2-GFP::HIS3*	Open Biosystems
YTH4515	*MAT*α *LEU2::GAL1/10_prom_*-*FKH1-TAP::HIS3*	This study (YTH4265×4269)
YTH4517	*LEU2::GAL1/10_prom_*-*FKH1-TAP::HIS3 LEU2::GAL1/10_prom_*-*FKH2-TAP::HIS3*	This study (YTH4515×4516)

### Media and methods

Media were prepared as previously described [28], [33]. Segregation of the *kanMX6* cassette was determined by patching spores onto 0.2 mg/ml geneticin-supplemented YPD plates. Mutants containing two or more *kanMX6* marked alleles were generated by selecting tetrads in which G418 resistance segregated in a 2∶2 fashion. *Escherichia coli* strains JM109 and DH10B were used to propagate DNA plasmids. DNA manipulations such as DNA minipreps, and yeast and *E. coli* transformations were carried out according to standard protocols [67]. Spot dilution assays were conducted by pipeting 3 µl of cells from samples generated from a 10-fold dilution series onto the various media shown and grown at the temperatures indicated. The starting spot generally contained 5×10^4^ cells. To assess resistance to oxidative stress, cultures were grown in 2% YPD at 30°C for 5 days and then diluted to an OD_600_ of 1 in depleted media (DM). Each of these cultures was divided into two samples and 100 mM H_2_O_2_ (EMD Chemicals) was added to one sample. Both samples were then incubated at 30°C for 1 hour. Viability was determined by plating diluted cells onto 2% YPD and comparing the growth of the H_2_O_2_ treated culture to that of the non-treated control culture. Clb2 stability was determined by growing the indicated cells to early log phase at 30°C, then adding 2 µg/ml α factor, when using *BAR1* strains, in media at pH 3.5 to arrest cells in G1. After 1.5 hours, another 2 µg/ml was added, with continued incubation for another 1 hour. After this treatment, cells were arrested in G1. Cell cycle arrest was confirmed by microscopic visualization of the cells and by flow cytometry. The α factor was then washed away and fresh media containing 10 µg/ml cycloheximide was added. The incubation was continued at 30°C, with samples removed every 15 minutes for Western analysis using antibodies against endogenous Clb2 and GAPDH. FACS was performed as described previously [28]. To characterize *fkh1Δ fkh2Δ* FACS profiles the following changes were made to overcome the persistent cell/cell contacts inherent to this mutant. 1 ml of culture (OD_600_ 0.4) was resuspended in 50 µl followed by the addition 10 µl of 12.5 U/µl lyticase. This solution was incubated for 15 minutes at room temperature, followed by the addition of 500 µl 50 mM Tris. The cells were then sonicated for 6 seconds at output 4. The cells were centrifuged, resuspended in 1 ml 70% EtOH and processed as usual past this step.

### Lifespan assays

Chronological lifespan was performed as previously described [29], [40], [41]. Briefly, overnight CM (2% glucose) cultures were diluted to OD_600_ 0.5 in fresh CM with a flask to culture volume ratio of 5∶1. The incubation continued (200 RPM) at 30°C. Each day the same volume of culture was diluted and plated to evaluate colony forming units (CFU) as a measure of viability. When the CFU counts peaked, this was deemed stationary phase and denoted as Day 1. Every two days CFU were determined and compared to Day 1. For severe caloric restriction (SCR) experiments, once stationary phase was reached in CM (Day1), cultures were washed and resuspended in sterile distilled H_2_O, with washes of equal volume of water every two to four days to remove metabolites produced by the cells. Galactose and hydrogen peroxide were added to appropriate cultures upon reaching Day 1 to final concentrations of 0.05% and 25 mM respectively.

For fluorescent localization, samples were obtained from CLS cultures, washed and mounted in PBS or Ultracruz mounting medium (Santa Cruz Biotechnology sc-24941) and imaged using 100× oil immersion with an Olympus BX51 fluorescent microscope. Images were captured using an INFINITY 3-1UM camera, and analyzed with Infinity Analyse software version 5.0.3 (Lumenera).

Replicative lifespan experiments were performed as previously described [29]. The plates were stored at 4°C each night. The experiments were performed blind; the genotypes of the strains and conditions used were not revealed to the experimenter until the final mother stopped producing daughters.

### Western analysis

Yeast whole cell protein extraction was performed as previously described [28]. Samples were resolved on a 15% acrylamide SDS-PAGE gel, which was stained with Coomassie brilliant blue R-250 (OmniPUR) or transferred directly to nitrocellulose membrane (PALL) at 400 mAmps for 1 hour. Protein loading was analyzed using ImageJ 1.37v software (NIH). Equalized samples were then transferred to nitrocellulose membrane. For Western analysis, the membranes were blocked in 5% non-fat milk (Biorad) and PBST overnight at 4°C. The membranes were then incubated with primary antibody in 5% non-fat milk and PBST for 1.5 hours at room temperature or overnight at 4°C. Rabbit polyclonal anti-H2B (Abcam), polyclonal anti-H3 (Abcam), and polyclonal anti-H4 (Abcam) were used at a 1∶4000 dilution. Rabbit polyclonal anti-Clb2 (Santa Cruz; Y-180) was used at 1∶2000. The TAP antibody (Open Biosystems) was used at a dilution of 1∶1000. Mouse monoclonal anti-GAPDH (Sigma) was used at a dilution of 1∶20,000. The membranes were then washed 3 times in PBST for 15 minutes, and incubated in 1∶10,000 dilution of secondary antibody in 5% non-fat milk and PBST for 1 hour at room temperature. After another 3 washes in PBST for 15 minutes, the membranes were processed with Enhanced Chemiluminescence reagent (PerkinElmer) and exposed to Kodak film.
